# Protecting against researcher bias in secondary data analysis: challenges and potential solutions

**DOI:** 10.1007/s10654-021-00839-0

**Published:** 2022-01-13

**Authors:** Jessie R. Baldwin, Jean-Baptiste Pingault, Tabea Schoeler, Hannah M. Sallis, Marcus R. Munafò

**Affiliations:** 1 Department of Clinical, Educational and Health Psychology, Division of Psychology and Language Sciences, University College London, London, WC1H 0AP UK; 2 Social, Genetic and Developmental Psychiatry Centre, Institute of Psychiatry, Psychology and Neuroscience, King’s College London, London, UK; 3 MRC Integrative Epidemiology Unit at the University of Bristol, Bristol Medical School, University of Bristol, Bristol, UK; 4 School of Psychological Science, University of Bristol, Bristol, UK; 5 Centre for Academic Mental Health, Population Health Sciences, University of Bristol, Bristol, UK; 6 NIHR Biomedical Research Centre, University Hospitals Bristol NHS Foundation Trust and University of Bristol, Bristol, UK

**Keywords:** Secondary data analysis, Pre-registration, Open science, Researcher bias

## Abstract

Analysis of secondary data sources (such as cohort studies, survey data, and administrative records) has the potential to provide answers to science and society’s most pressing questions. However, researcher biases can lead to questionable research practices in secondary data analysis, which can distort the evidence base. While pre-registration can help to protect against researcher biases, it presents challenges for secondary data analysis. In this article, we describe these challenges and propose novel solutions and alternative approaches. Proposed solutions include approaches to (1) address bias linked to prior knowledge of the data, (2) enable pre-registration of non-hypothesis-driven research, (3) help ensure that pre-registered analyses will be appropriate for the data, and (4) address difficulties arising from reduced analytic flexibility in pre-registration. For each solution, we provide guidance on implementation for researchers and data guardians. The adoption of these practices can help to protect against researcher bias in secondary data analysis, to improve the robustness of research based on existing data.

## Introduction

Secondary data analysis has the potential to provide answers to science and society’s most pressing questions. An abundance of secondary data exists—cohort studies, surveys, administrative data (e.g., health records, crime records, census data), financial data, and environmental data—that can be analysed by researchers in academia, industry, third-sector organisations, and the government. However, secondary data analysis is vulnerable to questionable research practices (QRPs) which can distort the evidence base. These QRPs include p-hacking (i.e., exploiting analytic flexibility to obtain statistically significant results), selective reporting of statistically significant, novel, or “clean” results, and hypothesising after the results are known (HARK-ing [i.e., presenting unexpected results as if they were predicted]; [1]. Indeed, findings obtained from secondary data analysis are not always replicable [2, 3], reproducible [4], or robust to analytic choices [5, 6]. Preventing QRPs in research based on secondary data is therefore critical for scientific and societal progress.

A primary cause of QRPs is common cognitive biases that affect the analysis, reporting, and interpretation of data [7–10]. For example, apophenia (the tendency to see patterns in random data) and confirmation bias (the tendency to focus on evidence that is consistent with one’s beliefs) can lead to particular analytical choices and selective reporting of “publishable” results [11–13]. In addition, hindsight bias (the tendency to view past events as predictable) can lead to HARK-ing, so that observed results appear more compelling.

The scope for these biases to distort research outputs from secondary data analysis is perhaps particularly acute, for two reasons. First, researchers now have increasing access to high-dimensional datasets that offer a multitude of ways to analyse the same data [6]. Such analytic flexibility can lead to different conclusions depending on the analytical choices made [5, 14, 15]. Second, current incentive structures in science reward researchers for publishing statistically significant, novel, and/or surprising findings [16]. This combination of opportunity and incentive may lead researchers—consciously or unconsciously—to run multiple analyses and only report the most “publishable” findings.

**Fig. 1:**
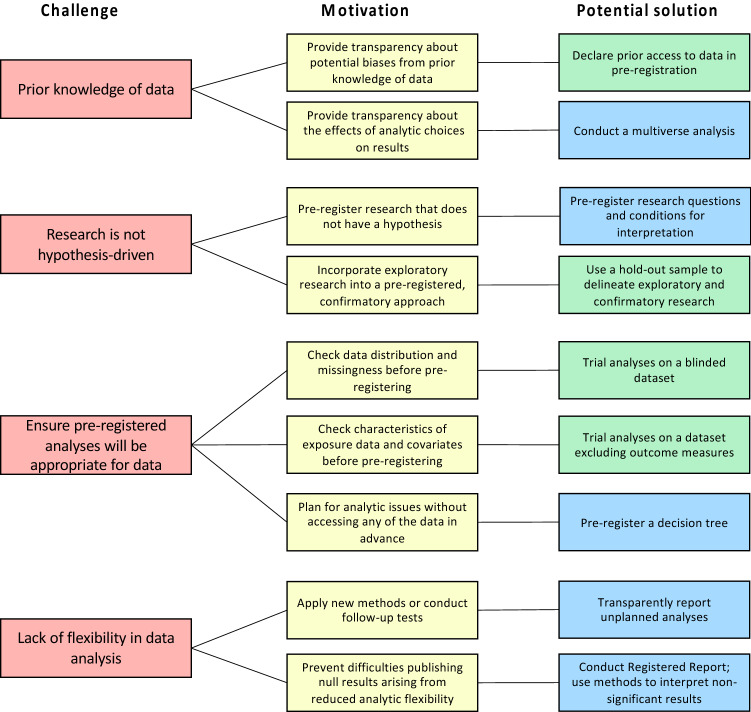
Challenges in pre-registering secondary data analysis and potential solutions (according to researcher motivations). *Note*: In the “Potential solution” column, blue boxes indicate solutions that are researcher-led; green boxes indicate solutions that should be facilitated by data guardians

One way to help protect against the effects of researcher bias is to pre-register research plans [17, 18]. This can be achieved by pre-specifying the rationale, hypotheses, methods, and analysis plans, and submitting these to either a third-party registry (e.g., the Open Science Framework [OSF]; https://osf.io/), or a journal in the form of a Registered Report [19]. Because research plans and hypotheses are specified before the results are known, pre-registration reduces the potential for cognitive biases to lead to p-hacking, selective reporting, and HARK-ing [20]. While pre-registration is not necessarily a panacea for preventing QRPs (Table 1), meta-scientific evidence has found that pre-registered studies and Registered Reports are more likely to report null results [21–23], smaller effect sizes [24], and be replicated [25]. Pre-registration is increasingly being adopted in epidemiological research [26, 27], and is even required for access to data from certain cohorts (e.g., the Twins Early Development Study [28]). However, pre-registration (and other open science practices; Table 2) can pose particular challenges to researchers conducting secondary data analysis [29], motivating the need for alternative approaches and solutions. Here we describe such challenges, before proposing potential solutions to protect against researcher bias in secondary data analysis (summarised in Fig. 1).

**Table 1:** Limitations in the use of pre-registration to address QRPs

Limitation	Example
Pre-registration may not prevent selective reporting/outcome switching	The COMPare Trials Project [62] assessed outcome switching in clinical trials published in the top 5 medical journals between October 2015 and January 2016. Among 67 clinical trials, on average, each trial reported 58.2% of its specified outcomes, and silently added 5.3 new outcomes
Pre-registration may be performed retrospectively after the results are known	Mathieu et al. [63] assessed 323 clinical trials published in 2008 in the top 10 medical journals. 45 trials (13.9%) were registered after the completion of the study
Deviations from pre-registered protocols are common	Claesen et al. [57] assessed all pre-registered articles published in Psychological Science and between February 2015 and November 2017. All 23 articles deviated from the pre-registration, and only one study disclosed the deviation
Pre-registration may not improve the credibility of hypotheses	Rubin [64] and Szollosi, Kellen [65] argue that formulating hypotheses post-hoc (HARK-ing) is not problematic if they are deduced from pre-existing theory or evidence, rather than induced from the current results

**Table 2:** Challenges and potential solutions regarding sharing pre-existing data

Challenge	Potential solutions
*Data cannot be made open*: Many datasets cannot be publicly shared because of ethical and legal requirements	Share a synthetic dataset (a simulated dataset which mimics an original dataset by preserving its statistical properties and associations between variables). For a tutorial, see Quintana [66]
	Provide specific instructions on how data can be accessed and links to codebooks/data dictionaries with variable information [67]
*Open data can lead to sequential testing problems:* If different researchers conduct similar statistical tests on a dataset and do not correct for multiple testing, this increases the risk of false positives [68]	Test whether findings replicate in independent samples, as the chance of two identical false positives occurring in independent samples is small
	Ensure that the research question is distinct from prior studies on the given dataset, to help ensure that proposed analyses are part of a different statistical family. Multiple analyses on a single dataset will not lead to false positives if the analyses are part of different statistical families

## Challenges of pre-registration for secondary data analysis

### Prior knowledge of the data

Researchers conducting secondary data analysis commonly analyse data from the same dataset multiple times throughout their careers. However, prior knowledge of the data increases risk of bias, as prior expectations about findings could motivate researchers to pursue certain analyses or questions. In the worst-case scenario, a researcher might perform multiple preliminary analyses, and only pursue those which lead to notable results (perhaps posting a pre-registration for these analyses, even though it is effectively post hoc). However, even if the researcher has *not* conducted specific analyses previously, they may be biased (either consciously or subconsciously) to pursue certain analyses after testing related questions with the same variables, or even by reading past studies on the dataset. As such, pre-registration cannot fully protect against researcher bias when researchers have previously accessed the data.

### Research may not be hypothesis-driven

Pre-registration and Registered Reports are tailored towards hypothesis-driven, confirmatory research. For example, the OSF pre-registration template requires researchers to state “specific, concise, and testable hypotheses”, while Registered Reports do not permit purely exploratory research [30], although a new Exploratory Reports format now exists [31]. However, much research involving secondary data is not focused on hypothesis testing, but is exploratory, descriptive, or focused on estimation—in other words, examining the magnitude and robustness of an association as precisely as possible, rather than simply testing a point null. Furthermore, without a strong theoretical background, hypotheses will be arbitrary and could lead to unhelpful inferences [32, 33], and so should be avoided in novel areas of research.

### Pre-registered analyses are not appropriate for the data

With pre-registration, there is always a risk that the data will violate the assumptions of the pre-registered analyses [17]. For example, a researcher might pre-register a parametric test, only for the data to be non-normally distributed. However, in secondary data analysis, the extent to which the data shape the appropriate analysis can be considerable. First, longitudinal cohort studies are often subject to missing data and attrition. Approaches to deal with missing data (e.g., listwise deletion; multiple imputation) depend on the characteristics of missing data (e.g., the extent and patterns of missingness [34]), and so pre-specifying approaches to dealing with missingness may be difficult, or extremely complex. Second, certain analytical decisions depend on the nature of the observed data (e.g., the choice of covariates to include in a multiple regression might depend on the collinearity between the measures, or the degree of missingness of different measures that capture the same construct). Third, much secondary data (e.g., electronic health records and other administrative data) were never collected for research purposes, so can present several challenges that are impossible to predict in advance [35]. These issues can limit a researcher’s ability to pre-register a precise analytic plan prior to accessing secondary data.

### Lack of flexibility in data analysis

Concerns have been raised that pre-registration limits flexibility in data analysis, including justifiable exploration [36–38]. For example, by requiring researchers to commit to a pre-registered analysis plan, pre-registration could prevent researchers from exploring novel questions (with a hypothesis-free approach), conducting follow-up analyses to investigate notable findings [39], or employing newly published methods with advantages over those pre-registered. While this concern is also likely to apply to primary data analysis, it is particularly relevant to certain fields involving secondary data analysis, such as genetic epidemiology, where new methods are rapidly being developed [40], and follow-up analyses are often required (e.g., in a genome-wide association study to further investigate the role of a genetic variant associated with a phenotype). However, this concern is perhaps over-stated – pre-registration does not *preclude* unplanned analyses; it simply makes it more transparent that these analyses are post hoc. Nevertheless, another understandable concern is that reduced analytic flexibility could lead to difficulties in publishing papers and accruing citations. For example, pre-registered studies are more likely to report null results [22, 23], likely due to reduced analytic flexibility and selective reporting. While this is a positive outcome for research integrity, null results are less likely to be published [13, 41, 42] and cited [11], which could disadvantage researchers’ careers.

## Solutions

In this section, we describe potential solutions to address the challenges involved in pre-registering secondary data analysis, including approaches to (1) address bias linked to prior knowledge of the data, (2) enable pre-registration of non-hypothesis-driven research, (3) ensure that pre-planned analyses will be appropriate for the data, and (4) address potential difficulties arising from reduced analytic flexibility.

### Challenge: Prior knowledge of the data

#### Declare prior access to data

To increase transparency about potential biases arising from knowledge of the data, researchers could routinely report all prior data access in a pre-registration [29]. This would ideally include evidence from an independent gatekeeper (e.g., a data guardian of the study) stating whether data and relevant variables were accessed by each co-author. To facilitate this process, data guardians could set up a central “electronic checkout” system that records which researchers have accessed data, what data were accessed, and when [43]. The researcher or data guardian could then provide links to the checkout histories for all co-authors in the pre-registration, to verify their prior data access. If it is not feasible to provide such objective evidence, authors could self-certify their prior access to the dataset and where possible, relevant variables—preferably listing any publications and in-preparation studies based on the dataset [29]. Of course, self-certification relies on trust that researchers will accurately report prior data access, which could be challenging if the study involves a large number of authors, or authors who have been involved on many studies on the dataset. However, it is likely to be the most feasible option at present as many datasets do not have available electronic records of data access. For further guidance on self-certifying prior data access when pre-registering secondary data analysis studies on a third-party registry (e.g., the OSF), we recommend referring to the template by Van den Akker, Weston [29].

The extent to which prior access to data renders pre-registration invalid is debatable. On the one hand, even if data have been accessed previously, pre-registration is likely to reduce QRPs by encouraging researchers to commit to a pre-specified analytic strategy. On the other hand, pre-registration does not fully protect against researcher bias where data have already been accessed, and can lend added credibility to study claims, which may be unfounded. Reporting prior data access in a pre-registration is therefore important to make these potential biases transparent, so that readers and reviewers can judge the credibility of the findings accordingly. However, for a more rigorous solution which *protects* against researcher bias in the context of prior data access, researchers should consider adopting a multiverse approach.

### Conduct a multiverse analysis

A multiverse analysis involves identifying all potential analytic choices that could justifiably be made to address a given research question (e.g., different ways to code a variable, combinations of covariates, and types of analytic model), implementing them all, and reporting the results [44]. Notably, this method differs from the traditional approach in which findings from only one analytic method are reported. It is conceptually similar to a sensitivity analysis, but it is far more comprehensive, as often hundreds or thousands of analytic choices are reported, rather than a handful. By showing the results from all defensible analytic approaches, multiverse analysis reduces scope for selective reporting and provides insight into the robustness of findings against analytical choices (for example, if there is a clear convergence of estimates, irrespective of most analytical choices). For causal questions in observational research, Directed Acyclic Graphs (DAGs) could be used to inform selection of covariates in multiverse approaches [45] (i.e., to ensure that confounders, rather than mediators or colliders, are controlled for).

Specification curve analysis [46] is a form of multiverse analysis that has been applied to examine the robustness of epidemiological findings to analytic choices [6, 47]. Specification curve analysis involves three steps: (1) identifying all analytic choices – termed “specifications”, (2) displaying the results graphically with magnitude of effect size plotted against analytic choice, and (3) conducting joint inference across all results. When applied to the association between digital technology use and adolescent well-being [6], specification curve analysis showed that the (small, negative) association diminished after accounting for adequate control variables and recall bias – demonstrating the sensitivity of results to analytic choices.

Despite the benefits of the multiverse approach in addressing analytic flexibility, it is not without limitations. First, because each analytic choice is treated as equally valid, including less justifiable models could bias the results away from the truth. Second, the choice of specifications can be biased by prior knowledge (e.g., a researcher may choose to omit a covariate to obtain a particular result). Third, multiverse analysis may not entirely prevent selective reporting (e.g., if the full range of results are not reported), although pre-registering multiverse approaches (and specifying analytic choices) could mitigate this. Last, and perhaps most importantly, multiverse analysis is technically challenging (e.g., when there are hundreds or thousands of analytic choices) and can be impractical for complex analyses, very large datasets, or when computational resources are limited. However, this burden can be somewhat reduced by tutorials and packages which are being developed to standardise the procedure and reduce computational time [see 48, 49].

### Challenge: Research may not be hypothesis-driven

#### Pre-register research questions and conditions for interpreting findings

Observational research arguably does not need to have a hypothesis to benefit from pre-registration. For studies that are descriptive or focused on estimation, we recommend pre-registering research questions, analysis plans, and criteria for interpretation. Analytic flexibility will be limited by pre-registering specific research questions and detailed analysis plans, while post hoc interpretation will be limited by pre-specifying criteria for interpretation [50]. The potential for HARK-ing will also be minimised because readers can compare the published study to the original pre-registration, where a-priori hypotheses were not specified.

Detailed guidance on how to pre-register research questions and analysis plans for secondary data is provided in Van den Akker’s [29] tutorial. To pre-specify conditions for interpretation, it is important to anticipate – as much as possible – all potential findings, and state how each would be interpreted. For example, suppose that a researcher aims to test a causal relationship between X and Y using a multivariate regression model with longitudinal data. Assuming that all potential confounders have been fully measured and controlled for (albeit a strong assumption) and statistical power is high, three broad sets of results and interpretations could be pre-specified. First, an association between X and Y that is similar in magnitude to the unadjusted association would be consistent with a causal relationship. Second, an association between X and Y that is attenuated after controlling for confounders would suggest that the relationship is partly causal and partly confounded. Third, a minimal, non-statistically significant adjusted association would suggest a lack of evidence for a causal effect of X on Y. Depending on the context of the study, criteria could also be provided on the threshold (or range of thresholds) at which the effect size would justify different interpretations [51], be considered practically meaningful, or the smallest effect size of interest for equivalence tests [52]. While researcher biases might still affect the pre-registered criteria for interpreting findings (e.g., toward over-interpreting a small effect size as meaningful), this bias will at least be transparent in the pre-registration.

### Use a holdout sample to delineate exploratory and confirmatory research

Where researchers wish to integrate exploratory research into a pre-registered, confirmatory study, a holdout sample approach can be used [18]. Creating a holdout sample refers to the process of randomly splitting the dataset into two parts, often referred to as ‘training’ and ‘holdout’ datasets. To delineate exploratory and confirmatory research, researchers can first conduct exploratory data analysis on the training dataset (which should comprise a moderate fraction of the data, e.g., 35% [53]. Based on the results of the discovery process, researchers can pre-register hypotheses and analysis plans to formally test on the holdout dataset. This process has parallels with cross-validation in machine learning, in which the dataset is split and the model is developed on the training dataset, before being tested on the test dataset. The approach enables a flexible discovery process, before formally testing discoveries in a non-biased way.

When considering whether to use the holdout sample approach, three points should be noted. First, because the training dataset is not reusable, there will be a reduced sample size and loss of power relative to analysing the whole dataset. As such, the holdout sample approach will only be appropriate when the original dataset is large enough to provide sufficient power in the holdout dataset. Second, when the training dataset is used for exploration, subsequent confirmatory analyses on the holdout dataset may be overfitted (due to both datasets being drawn from the same sample), so replication in independent samples is recommended. Third, the holdout dataset should be created by an independent data manager or guardian, to ensure that the researcher does not have knowledge of the full dataset. However, it is straightforward to randomly split a dataset into a holdout and training sample and we provide example R code at: https://github.com/jr-baldwin/Researcher_Bias_Methods/blob/main/Holdout_script.md.

### Challenge: Pre-registered analyses are not appropriate for the data

#### Use blinding to test proposed analyses

One method to help ensure that pre-registered analyses will be appropriate for the data is to trial the analyses on a blinded dataset [54], before pre-registering. Data blinding involves obscuring the data values or labels prior to data analysis, so that the proposed analyses can be trialled on the data without observing the actual findings. Various types of blinding strategies exist [54], but one method that is appropriate for epidemiological data is “data scrambling” [55]. This involves randomly shuffling the data points so that any associations between variables are obscured, whilst the variable distributions (and amounts of missing data) remain the same. We provide a tutorial for how to implement this in R (see https://github.com/jr-baldwin/Researcher_Bias_Methods/blob/main/Data_scrambling_tutorial.md). Ideally the data scrambling would be done by a data guardian who is independent of the research, to ensure that the main researcher does not access the data prior to pre-registering the analyses. Once the researcher is confident with the analyses, the study can be pre-registered, and the analyses conducted on the unscrambled dataset.

Blinded analysis offers several advantages for ensuring that pre-registered analyses are appropriate, with some limitations. First, blinded analysis allows researchers to directly check the distribution of variables and amounts of missingness, without having to make assumptions about the data that may not be met, or spend time planning contingencies for every possible scenario. Second, blinded analysis prevents researchers from gaining insight into the potential findings prior to pre-registration, because associations between variables are masked. However, because of this, blinded analysis does not enable researchers to check for collinearity, predictors of missing data, or other covariances that may be necessary for model specification. As such, blinded analysis will be most appropriate for researchers who wish to check the data distribution and amounts of missingness before pre-registering.

### Trial analyses on a dataset excluding the outcome

Another method to help ensure that pre-registered analyses will be appropriate for the data is to trial analyses on a dataset excluding outcome data. For example, data managers could provide researchers with part of the dataset containing the exposure variable(s) plus any covariates and/or auxiliary variables. The researcher can then trial and refine the analyses ahead of pre-registering, without gaining insight into the main findings (which require the outcome data). This approach is used to mitigate bias in propensity score matching studies [26, 56], as researchers use data on the exposure and covariates to create matched groups, prior to accessing any outcome data. Once the exposed and non-exposed groups have been matched effectively, researchers pre-register the protocol ahead of viewing the outcome data. Notably though, this approach could help researchers to identify and address other analytical challenges involving secondary data. For example, it could be used to check multivariable distributional characteristics, test for collinearity between multiple predictor variables, or identify predictors of missing data for multiple imputation.

This approach offers certain benefits for researchers keen to ensure that pre-registered analyses are appropriate for the observed data, with some limitations. Regarding benefits, researchers will be able to examine associations between variables (excluding the outcome), unlike the data scrambling approach described above. This would be helpful for checking certain assumptions (e.g., collinearity or characteristics of missing data such as whether it is missing at random). In addition, the approach is easy to implement, as the dataset can be initially created without the outcome variable, which can then be added after pre-registration, minimising burden on data guardians. Regarding limitations, it is possible that accessing variables in advance could provide some insight into the findings. For example, if a covariate is known to be highly correlated with the outcome, testing the association between the covariate and the exposure could give some indication of the relationship between the exposure and the outcome. To make this potential bias transparent, researchers should report the variables that they already accessed in the pre-registration. Another limitation is that researchers will not be able to identify analytical issues relating to the outcome data in advance of pre-registration. Therefore, this approach will be most appropriate where researchers wish to check various characteristics of the exposure variable(s) and covariates, rather than the outcome. However, a “mixed” approach could be applied in which outcome data is provided in scrambled format, to enable researchers to also assess distributional characteristics of the outcome. This would substantially reduce the number of potential challenges to be considered in pre-registered analytical pipelines.

### Pre-register a decision tree

If it is not possible to access any of the data prior to pre-registering (e.g., to enable analyses to be trialled on a dataset that is blinded or missing outcome data), researchers could pre-register a decision tree. This defines the sequence of analyses and rules based on characteristics of the observed data [17]. For example, the decision tree could specify testing a normality assumption, and based on the results, whether to use a parametric or non-parametric test. Ideally, the decision tree should provide a contingency plan for each of the planned analyses, if assumptions are not fulfilled. Of course, it can be challenging and time consuming to anticipate every potential issue with the data and plan contingencies. However, investing time into pre-specifying a decision tree (or a set of contingency plans) could save time should issues arise during data analysis, and can reduce the likelihood of deviating from the pre-registration.

### Challenge: Lack of flexibility in data analysis

#### Transparently report unplanned analyses

Unplanned analyses (such as applying new methods or conducting follow-up tests to investigate an interesting or unexpected finding) are a natural and often important part of the scientific process. Despite common misconceptions, pre-registration does not permit such unplanned analyses from being included, as long as they are transparently reported as post-hoc. If there are methodological deviations, we recommend that researchers should (1) clearly state the reasons for using the new method, and (2) if possible, report results from both methods, to ideally show that the change in methods was not due to the results [57]. This information can either be provided in the manuscript or in an update to the original pre-registration (e.g., on the third-party registry such as the OSF), which can be useful when journal word limits are tight. Similarly, if researchers wish to include additional follow-up analyses to investigate an interesting or unexpected finding, this should be reported but labelled as “exploratory” or “post-hoc” in the manuscript.

### Ensure a paper’s value does not depend on statistically significant results

Researchers may be concerned that reduced analytic flexibility from pre-registration could increase the likelihood of reporting null results [22, 23], which are harder to publish [13, 42]. To address this, we recommend taking steps to ensure that the value and success of a study does not depend on a significant p-value. First, methodologically strong research (e.g., with high statistical power, valid and reliable measures, robustness checks, and replication samples) will advance the field, whatever the findings. Second, methods can be applied to allow for the interpretation of statistically non-significant findings (e.g., Bayesian methods [58] or equivalence tests, which determine whether an observed effect is surprisingly small [52, 59, 60]. This means that the results will be informative whatever they show, in contrast to approaches relying solely on null hypothesis significance testing, where statistically non-significant findings cannot be interpreted as meaningful. Third, researchers can submit the proposed study as a Registered Report, where it will be evaluated before the results are available. This is arguably the strongest way to protect against publication bias, as in-principle study acceptance is granted without any knowledge of the results. In addition, Registered Reports can improve the methodology, as suggestions from expert reviewers can be incorporated into the pre-registered protocol.

## Conclusion

Under a system that rewards novel and statistically significant findings, it is easy for subconscious human biases to lead to QRPs. However, researchers, along with data guardians, journals, funders, and institutions, have a responsibility to ensure that findings are reproducible and robust. While pre-registration can help to limit analytic flexibility and selective reporting, it involves several challenges for epidemiologists conducting secondary data analysis. The approaches described here aim to address these challenges (Fig. 1), to either improve the efficacy of pre-registration or provide an alternative approach to address analytic flexibility (e.g., a multiverse analysis). The responsibility in adopting these approaches should not only fall on researchers’ shoulders; data guardians also have an important role to play in recording and reporting access to data, providing blinded datasets and hold-out samples, and encouraging researchers to pre-register and adopt these solutions as part of their data request. Furthermore, wider stakeholders could incentivise these practices; for example, journals could provide a designated space for researchers to report deviations from the pre-registration, and funders could provide grants to establish best practice at the cohort level (e.g., data checkout systems, blinded datasets). Ease of adoption is key to ensure wide uptake, and we therefore encourage efforts to evaluate, simplify and improve these practices. Steps that could be taken to evaluate these practices are presented in Box 1.

More broadly, it is important to emphasise that researcher biases do not operate in isolation, but rather in the context of wider publication bias and a “publish or perish” culture. These incentive structures not only promote QRPs [61], but also discourage researchers from pre-registering and adopting other time-consuming reproducible methods. Therefore, in addition to targeting bias at the individual researcher level, wider initiatives from journals, funders, and institutions are required to address these institutional biases [7]. Systemic changes that reward rigorous and reproducible research will help researchers to provide unbiased answers to science and society’s most important questions.

## Box 1. Evaluation of approaches

To evaluate, simplify and improve approaches to protect against researcher bias in secondary data analysis, the following steps could be taken.

## Co-creation workshops to refine approaches

To obtain feedback on the approaches (including on any practical concerns or feasibility issues) co-creation workshops could be held with researchers, data managers, and wider stakeholders (e.g., journals, funders, and institutions).

## Empirical research to evaluate efficacy of approaches

To evaluate the effectiveness of the approaches in preventing researcher bias and/or improving pre-registration, empirical research is needed. For example, to test the extent to which the multiverse analysis can reduce selective reporting, comparisons could be made between effect sizes from multiverse analyses versus effect sizes from meta-analyses (of non-pre-registered studies) addressing the same research question. If smaller effect sizes were found in multiverse analyses, it would suggest that the multiverse approach can reduce selective reporting. In addition, to test whether providing a blinded dataset or dataset missing outcome variables could help researchers develop an appropriate analytical protocol, researchers could be randomly assigned to receive such a dataset (or no dataset), prior to pre-registration. If researchers who received such a dataset had fewer eventual deviations from the pre-registered protocol (in the final study), it would suggest that this approach can help ensure that proposed analyses are appropriate for the data.

## Pilot implementation of the measures

To assess the practical feasibility of the approaches, data managers could pilot measures for users of the dataset (e.g., required pre-registration for access to data, provision of datasets that are blinded or missing outcome variables). Feedback could then be collected from researchers and data managers via about the experience and ease of use.
